# Free Floating Right Heart Thrombus Associated with Acute Pulmonary Embolism: An Unsettled Therapeutic Difficulty

**DOI:** 10.1155/2015/364780

**Published:** 2015-05-11

**Authors:** Clovis Nkoke, Olivier Faucher, Lise Camus, Laurence Flork

**Affiliations:** ^1^Department of Internal Medicine and Specialties, University of Yaounde 1, Yaounde, Cameroon; ^2^Departement of Cardiology, Centre Hospitalier Guy Thomas, BP Box 167, 63204 Riom Cedex, France

## Abstract

Free floating right heart thrombus is a rare phenomenon in the context of acute pulmonary embolism and it is associated with a poor outcome. The increased use of echocardiography has led to an increased detection of right heart thrombi. However, optimal management of free floating right heart thrombus remains controversial with no clear consensus. We present the case of a 74-year-old woman who presented to the emergency department with acute onset dyspnea on minimal exertion which had developed over a period of 1 day. A computed tomography of the chest demonstrated massive bilateral proximal pulmonary embolism. A bedside transthoracic echocardiography performed showed a moderately dilated, poorly functioning right ventricle with visible highly mobile serpiginous thrombus moving to and fro across the tricuspid valve. Thrombolytic therapy was immediately initiated with tenecteplase which resulted in excellent results. Although there is no clear consensus for the management of right heart thrombus associated with pulmonary embolism, thrombolysis is readily available and can be effective in carefully selected patients.

## 1. Introduction

Free floating right heart thrombus-in-transit is a rare phenomenon in the context of acute pulmonary embolism and is most commonly encountered in hemodynamically unstable patients with shorter duration of symptoms [1]. The increased use of echocardiography has led to an increase in the detection of free floating right heart thrombi. Though the actual incidence is unknown, echocardiographic studies in patients with pulmonary embolism show an incidence of 7 to 18% [2].

Right heart thrombus is considered to be an extreme therapeutic emergency as it is associated with worse outcomes since they are an indication of imminent and potentially fatal pulmonary embolism [3–5]. The overall mortality rate in patients with right heart thrombus has been reported as 28% and as high as 100% in untreated patients [5].

Despite advances in detection, the optimal therapy for right heart thrombus-in-transit still remains a therapeutic dilemma because prospective randomized controlled studies are scarce. Existing published reports differ in their recommendations for treatment by advocating surgical removal, administration of thrombolytic agents, anticoagulation therapy with heparin, or using interventional percutaneous thrombus retrieval techniques. However, some report points to a better outcome with thrombolysis [2, 6].

## 2. Case Presentation

A 74-year-old patient was referred to our emergency department because of 1-day history of sudden onset dyspnea on minimal exertion that was preceded a couple of days earlier by right calf pain. There was neither chest pain nor syncope. Her past medical history was remarkable for hypertension and a right popliteal cyst diagnosed four years back. On physical examination she had a temperature of 36.8°C, a blood pressure of 109/70 mmHg, a heart rate of 110/minute, a respiratory rate of 32 cycles/minute, and hypoxemia at room air (oxygen saturation at 87%). There were no signs of right ventricular failure. There was tenderness of the right calf muscles with a palpable mass.

**Figure 1:**
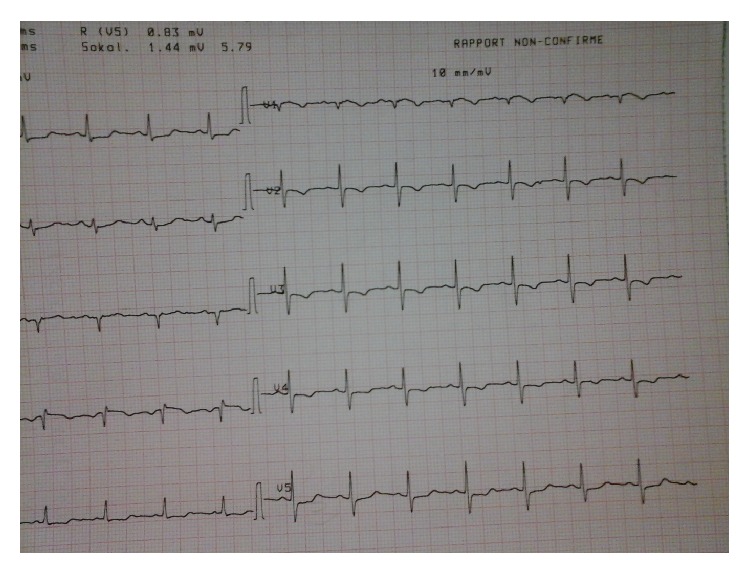
12-lead electrocardiography showing T wave inversion in the right precordial leads. There is ST elevation in AVR and V1 and ST depression in inferior leads: Treppe effect.

**Figure 2:**
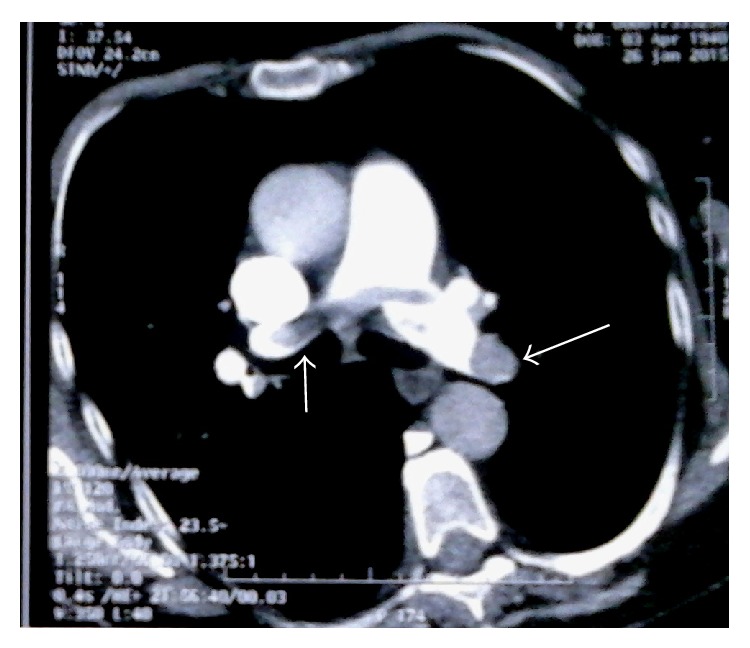
Computed tomography of the chest showing bilateral proximal pulmonary emboli (white arrows).

**Figure 3:**
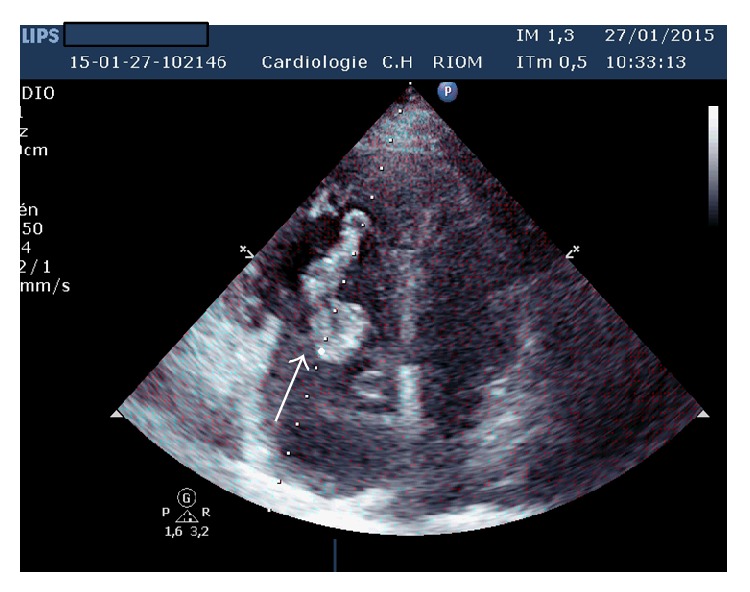
Apical four chamber view showing a highly mobile serpiginous thrombus in the right heart chambers (white arrow).

**Figure 4:**
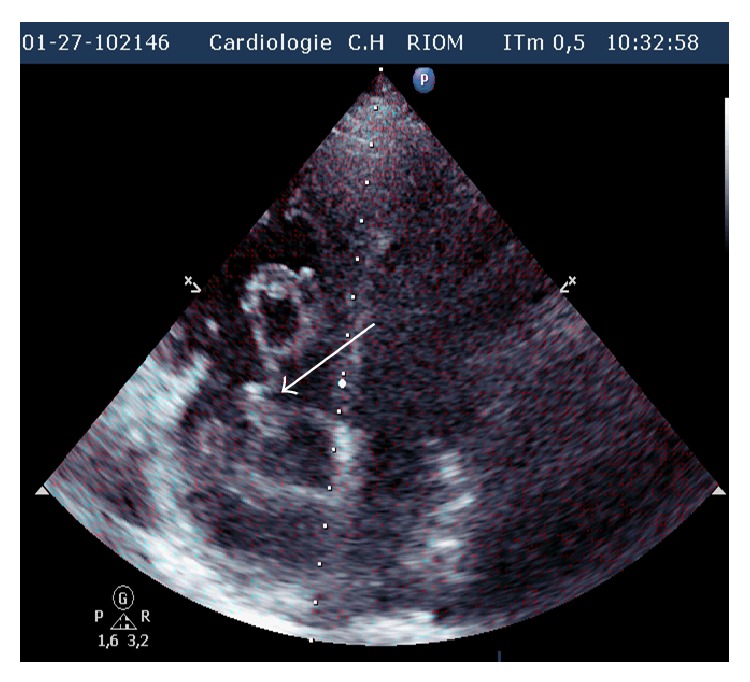
Apical four chamber view showing a serpiginous thrombus (white arrow) in the right heart chambers.

**Figure 5:**
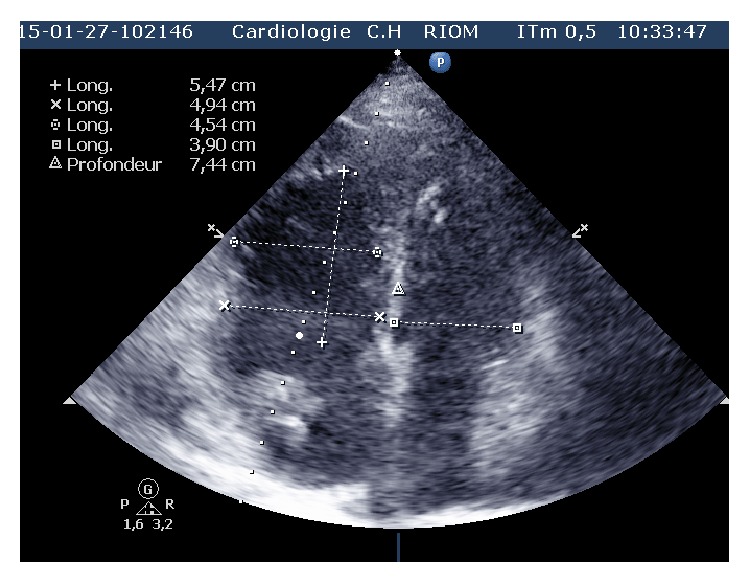
Apical four chamber view showing right ventricular dimensions (basal right ventricular diameter = 4.94 cm, mid-right ventricular diameter = 4.54 cm).

**Figure 6:**
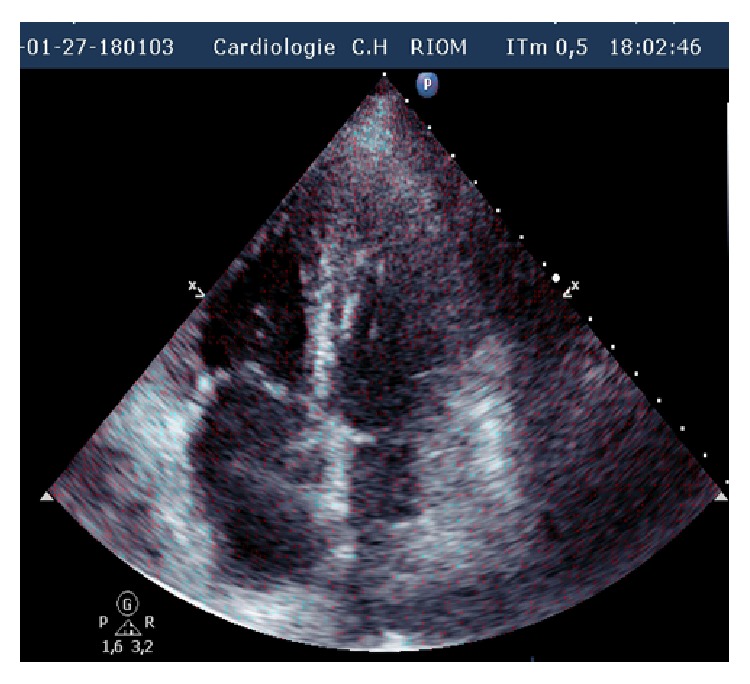
Apical four chamber view after thrombolysis showing complete disappearance of right heart thrombus.

Laboratory studies showed an elevated D-dimer >20 *μ*g/mL (*N* < 0.5), troponin at 1.819 *μ*g/L (*N* < 0.050), and NT proBNP at 6693 pg/mL (*N* < 1800 pg/mL for age >75 years). The chest radiograph was unremarkable. A 12-lead electrocardiogram demonstrated left axis deviation and T wave inversion in the right precordial leads suggesting acute right ventricular strain (Figure 1). There was ST elevation in AVR and V1 and ST depression in inferior leads: Treppe effect. A computed tomography of the chest showed massive bilateral proximal pulmonary embolism without pulmonary infarction (Figure 2). She was started on anticoagulation therapy with unfractionated heparin. A bedside transthoracic echocardiography performed showed a large highly mobile serpiginous like thrombus moving to and fro across the tricuspid valve (Figures 3 and 4). The attachment of the thrombus could not be ascertained. The right ventricle was moderately (Figure 5) dilated with decreased systolic function (an increased RV-LV diameter ratio, basal right ventricular diameter = 4.94 cm, mid-right ventricular diameter = 4.54 cm, tricuspid annulus plane systolic excursion = 11.5 mm, and peak systolic velocity at the tricuspid annulus = 7.01 cm/s). The pulmonary artery systolic pressure was 46 mmHg. There was no right to left shunting. The inferior vena cava was free of thrombus. The patient received immediate thrombolytic treatment with the recombinant tissue plasminogen activator, tenecteplase, followed by unfractionated heparin. A transthoracic echocardiography done six hours later showed a complete disappearance of the right heart thrombus (Figure 6) with improvement of right ventricular function and decrease in pulmonary artery systolic pressure to 23 mmHg. The patient did not show any signs of recurrent pulmonary embolism and hemodynamic deterioration. She presented mild hematuria that regressed over couple of days. A Doppler ultrasound of the lower limbs demonstrated thrombosis of the right popliteal vein. The patient was discharged home on oral anticoagulation with regular follow-up in outpatient clinic and lifelong anticoagulation was advised.

## 3. Discussion

Right heart thrombi may develop within the right heart chambers (type B) or they may be peripheral venous clots that accidentally lodge in the right heart on their way to the lungs (type A), known as right heart thrombi-in-transit. Type A thrombi have a worm-like shape and are extremely mobile. Type B thrombi are morphologically similar to left heart thrombi, are less mobile, attach to the right atrial or ventricular wall, and have a broad based attachment indicating that these develop within the right heart [3]. Our patient had a serpiginous thrombus moving to and fro into the right ventricle favoring type A thrombi. As demonstrated in previous studies of patients with pulmonary embolism with right heart thrombi, our patient had right ventricular dysfunction and elevation in cardiac troponin reflecting right ventricular strain and ischemia [2, 5, 7].

The finding of right ventricular thrombi in pulmonary embolism is associated with a higher mortality rate compared to pulmonary embolism without right heart thrombi. Rose et al. in a meta-analysis reported a mortality rate of up to 27%, while de Vrey et al. reported a mortality of >44% [5, 8].

Free floating right heart thrombus warrants immediate therapeutic intervention and any delay in treatment can lead to a fatal outcome [4]. Recommendations for treatment include anticoagulation therapy with heparin, administration of thrombolytic agents, or surgical removal of the thrombus [9]. Although it is associated with high mortality, there is no clear consensus on its management because prospective randomized controlled studies are scarce [2, 9]. A report by Chartier et al. pointed that there was no significant difference between these therapeutic approaches in terms of in-hospital mortality [2]. Thrombolysis is a simple and fast treatment option with numerous advantages including acceleration of pulmonary reperfusion, reduction in pulmonary hypertension, improvement of right ventricular function, and possibility of dissolving the intracardiac thrombus, pulmonary embolism, and the venous thromboembolism at the same time [10, 11]. However, the possibility of the clot breaking loose and embolization to the lungs where there is already a thrombus and bleeding might be problematic. Torbicki et al. [1] showed that the favorable result after thrombolysis could be related to the shorter delay between the presumed onset of symptoms and hospitalization in patients where pulmonary embolism was associated with mobile clots in the right heart (2.2 versus 4.5 days). Ferrari et al. [12] showed that after thrombolysis, 50% of the clots disappeared within 2 hours, whereas the remainder disappeared within 12–24 hrs. This delayed disappearance of the thrombi supports the decision to defer surgery after thrombolysis until at least 24 hours. Anticoagulation with heparin is more antithrombotic than a thrombolytic agent. It is inadequate as the sole treatment of a threatening recurrent pulmonary embolism to an already compromised pulmonary circulation. Surgical embolectomy with exploration of the right heart chambers and pulmonary arteries under cardiopulmonary bypass is another treatment option and it is the preferred treatment in hemodynamically unstable patients [2], particularly for cases in which thrombolysis is contraindicated or if thrombolysis is ineffective; but it is not readily available in many centers and it is sometimes associated with high mortality [2]. A recent review has suggested that meticulous surgical technique has significantly lowered the mortality associated with this procedure and can be extended to include hemodynamically stable patients [13].

## 4. Conclusion

Free floating right heart thrombi are rare in the context of acute pulmonary embolism. They are associated with a high mortality and as such represent a therapeutic emergency. Echocardiography is essential in their diagnosis. Optimal treatment is yet to be determined but thrombolysis is readily available and effective.
